# Bilateral upper extremity motor priming (BUMP) plus task-specific training for severe, chronic upper limb hemiparesis: study protocol for a randomized clinical trial

**DOI:** 10.1186/s13063-022-06465-9

**Published:** 2022-06-22

**Authors:** Mary Ellen Stoykov, Olivia M. Biller, Alexandra Wax, Erin King, Jacob M. Schauer, Louis F. Fogg, Daniel M. Corcos

**Affiliations:** 1 Arms & Hands Lab, Shirley Ryan AbilityLab, Chicago, IL USA; 2 Department of Physical Medicine and Rehabilitation, Feinberg School of Medicine, Northwestern University, Chicago, IL USA; 3 Department of Occupational Therapy, Jefferson College of Rehabilitation Sciences, Thomas Jefferson University, Philadelphia, PA USA; 4 Think & Speak Lab, Arms & Hands Lab, Shirley Ryan AbilityLab, Chicago, USA; 5 Interdepartmental Institution of Neuroscience, Northwestern University, Chicago, USA; 6 Department of Preventive Medicine – Division of Biostatistics, Feinberg School of Medicine, Northwestern University, Chicago, USA; 7 Department of Occupational Therapy, College of Applied Health Sciences, University of Illinois at Chicago, Chicago, USA; 8 Department of Physical Therapy and Human Movement Sciences, Feinberg School of Medicine, Northwestern University, Chicago, USA

**Keywords:** Chronic stroke, Priming, Task-specific training, Transcranial magnetic stimulation, Upper limb rehabilitation

## Abstract

**Background:**

Various priming techniques to enhance neuroplasticity have been examined in stroke rehabilitation research. Most priming techniques are costly and approved only for research. Here, we describe a priming technique that is cost-effective and has potential to significantly change clinical practice. Bilateral motor priming uses the Exsurgo priming device (Exsurgo Rehabilitation, Auckland, NZ) so that the less affected limb drives the more affected limb in bilateral symmetrical wrist flexion and extension. The aim of this study is to determine the effects of a 5-week protocol of bilateral motor priming in combination with task-specific training on motor impairment of the affected limb, bimanual motor function, and interhemispheric inhibition in moderate to severely impaired people with stroke.

**Methods:**

Seventy-six participants will be randomized to receive either 15, 2-h sessions, 3 times per week for 5 weeks (30 h of intervention) of bilateral motor priming and task-specific training (experimental group) or the same dose of control priming plus the task-specific training protocol. The experimental group performs bilateral symmetrical arm movements via the Exsurgo priming device which allows both wrists to move in rhythmic, symmetrical wrist flexion and extension for 15 min. The goal is one cycle (wrist flexion and wrist extension) per second. The control priming group receives transcutaneous electrical stimulation below sensory threshold for 15 min prior to the same 45 min of task-specific training. Outcome measures are collected at pre-intervention, post-intervention, and follow-up (8 weeks post-intervention). The primary outcome measure is the Fugl-Meyer Test of Upper Extremity Function. The secondary outcome is the Chedoke Arm and Hand Activity Index-Nine, an assessment of bimanual functional tasks.

**Discussion:**

To date, there are only 6 studies documenting the efficacy of priming using bilateral movements, 4 of which are pilot or feasibility studies. This is the first large-scale clinical trial of bilateral priming plus task-specific training. We have previously completed a feasibility intervention study of bilateral motor priming plus task-specific training and have considerable experience using this protocol.

**Trial registration:**

ClinicalTrials.gov NCT03517657. Retrospectively registered on May 7, 2018.

**Supplementary Information:**

The online version contains supplementary material available at 10.1186/s13063-022-06465-9.

## Administrative information

Note: the numbers in curly brackets in this protocol refer to SPIRIT checklist item numbers. The order of the items has been modified to group similar items (see http://www.equator-network.org/reporting-guidelines/spirit-2013-statement-defining-standard-protocol-items-for-clinical-trials/).

**Table:** 

Title {1}	Bilateral upper extremity motor priming (BUMP) plus task specific training for severe, chronic upper limb hemiparesis: Study protocol for a randomized clinical trial
Trial registration {2a and 2b}	ClinicalTrials.gov, ID: NCT03517657. Registered on May 7^th^, 2018.
Protocol version {3}	Version 14, July 14, 2021
Funding {4}	This study is funded by grants from the NIH (1RO1HD091492-03) and NUCATS. The original funding documentation is contained in an Additional file (see Additional file 1).
Author details {5a}	Mary Ellen Stoykov, PhD, OTR/L, Arms & Hands Lab, Shirley Ryan AbilityLab Department of Physical Medicine and Rehabilitation^2^ Feinberg School of Medicine Northwestern University Olivia M. Biller, OTD Department of Occupational Therapy Jefferson College of Rehabilitation Sciences Thomas Jefferson University Alexandra Wax, MS, OTR/L Think & Speak Lab, Arms & Hands Lab Shirley Ryan AbilityLab Erin King, MS, OTR/L Interdepartmental Institution of Neuroscience Northwestern University Jacob M. Schauer, PhD Department of Preventive Medicine – Division of Biostatistics Feinberg School of Medicine Northwestern University Louis F. Fogg, PhD Department of Occupational Therapy University of Illinois at Chicago Daniel M. Corcos, PhD Department of Physical Therapy and Human Movement Sciences Feinberg School of Medicine Northwestern University
Name and contact information for the trial sponsor {5b}	Northwestern University 633 Clark Street Evanston, IL 60208
Role of sponsor {5c}	All aspects of this study including trial design, methods of data collection, statistical analysis, interpretation of results, scientific writing, and dissemination are conducted independently from the study sponsor.

## Introduction

### Background and rationale {6a}

The decline in stroke mortality over the twentieth century [1] has increased incidence of post-stroke disability, and the most common disability in the stroke population is upper extremity (UE) hemiparesis. Constraint-induced movement therapy (CIMT) is an effective intervention but is only appropriate for stroke survivors with mild UE impairment who are in the upper quartile of residual function [2]. Thus, alternative treatments are needed to target stroke survivors with more moderate and severe impairments of the UE, whose prognosis for motor recovery is less favorable.

In an observational study, Ward and colleagues [3] demonstrated a large improvement in the Fugl-Meyer Test of Upper Extremity Function (FMUE) median score (FMUEΔ 8.0, IQR=4–11) in severely impaired individuals after 90 h of various types of occupational therapy. While the improvement was impressive, the study did not inform about the superiority of any specific training. Also, 90 h of training is a very large dose that is not sustainable in the current healthcare climate in the USA. Other studies using unilateral training for individuals with severe UE impairment have shown improvements that can be described as modest at best including robotic training (FMUEΔ = 1.11 ± 1.01) [4]; unilateral task-specific training in an active comparator group (FMUEΔ = 3.1 ± 5.3) [5]; and task-specific training + robotics (FMUEΔ = +3.25 ± 1.68) [6]. These studies did not demonstrate an improvement in the FMUE of ≥ 4.25 which is the estimated clinically important difference [7]. More impaired individuals may need either a larger dose or an augmentative intervention.

Motor priming is a construct used to describe a variety of techniques that optimize the brain’s response to subsequent training and may enhance neuroplasticity and motor performance [8–10]. Shiner et al. [11] compared bilateral motor priming (BMP) plus Wii therapy to Wii therapy alone in subacute and chronic stroke subjects. The result was in favor of the bilateral priming plus Wii training group that, at follow-up, had a significantly greater mean FMUE [12] score than with Wii training alone (6.3 between-group difference) [11]. There was a large range of impairment levels in the Shiner et al. study, and those individuals with more severe impairment had the largest improvement. This result suggests that BMP may magnify improvements inherent in therapy protocols and facilitate sustained improvements over time in individuals with severe UE impairment. Stoykov and colleagues [13] used a task-specific training (TST) protocol and combined it with either BMP or stroke education (control). The bilateral priming group had a substantial increase in FMUE scores from pre-intervention to follow-up (FMUEΔ = 10 ± 6.1) while the improvement in the control group was modest (FMUEΔ = 3.56 ± 4.1.) These data were used as the pilot data for the grant submission.

Another priming + TST study examining UE hemiparesis in severely impaired participants used sensory-based priming plus TST [14] compared to TST alone, and the between-group differential of the FMUE was highest at follow-up (between-group FMUEΔ was 4.4 ± 1.1). This finding is consistent with other studies confirming that the largest difference between priming and control group is at follow-up [13–18].

This clinical trial examines the use of BMP, a non-invasive, cost-effective neuromodulation technique. BMP consists of continuous, bilateral wrist flexion and extension using a device with a mechanical linkage so that the less affected hand and the more affected one move in symmetry [19]. This study is a pivotal step towards developing and using a practical neuromodulatory technique to prime the central nervous system to respond with greater efficacy to behavioral interventions for people with moderate to severe UE hemiparesis. In addition to the benefits mentioned above and compared to priming using more invasive methods such as repetitive transcranial magnetic stimulation (rTMS), BMP is (1) cost-effective; (2) available to a larger pool of people due to the absence of safety concerns; (3) does not require a skilled operator; and (4) can potentially be implemented into the clinic [20]. There are no known risks to bilateral priming.

### Objectives {7}

The purpose of this study is to test the hypothesis that bilateral symmetrical arm movements prime cortical regions and enhance neuroplasticity as measured by behavioral and cortical measures. Specifically, this trial will address two main objectives and test their associated hypotheses. The primary objective is to determine the magnitude of change in upper limb function and impairment in chronic stroke survivors who have undergone 30 h of BMP + TST. Primary hypothesis 1.1 is that the combination of BMP + TST will produce a between-group difference in improvement on the FMUE of at least 6.0 points more than control priming (CP) + TST at the follow-up timepoint (8 weeks post-treatment cessation). Secondary hypothesis 1.2 is that the combination of BMP + TST will increase scores on the Chedoke Arm & Hand Activity Index (CAHAI-9) by 3 points more than CP + TST, 8 weeks after the post-test (follow-up).

The secondary objective is to determine the effects of bilateral priming on cortical mechanisms measured by TMS. Hypothesis 2.1 is that BMP + TST will increase TCI from ipsilesional to contralesional hemisphere at post-treatment (following 30 h of treatment) and at 8 weeks after treatment cessation (follow-up), but there will be no change in the CP + TST group. Hypothesis 2.2 is that an increase in ipsilesional TCI will be positively associated with changes in the FMUE. We will perform a correlation analysis to test this relationship.

### Trial design {8}

This protocol adheres to the guidance of the Standard Protocol Items: Recommendations for Interventional Trials (SPIRIT) statement for reporting randomized clinical trials [21]. The study design is a stratified, randomized, masked, and parallel, two-arm intervention study of the effects of BMP and TST. This is a two-site superiority trial.

## Methods: participants, interventions, and outcomes

### Study setting {9}

Assessments are performed at the Northwestern University Department of Physical Therapy and Human Movement Science (PTHMS). Prior to the COVID-19 pandemic, treatment intervention and some of the assessments occurred at the Shirley Ryan AbilityLab. Northwestern University human subjects’ research was closed in March of 2020. Research resumed in July of 2020 but with significant restrictions specifying number of participants per research lab and strict adherence to protective equipment for both investigators and participants. Due to these restrictions, and preferences of the study team, the investigators decided to use both the Northwestern PTHMS Department and Shirley Ryan AbilityLab for treatment. Assessments are now only performed at Northwestern PTHMS Department. Both locations are in Chicago, Illinois.

### Eligibility criteria {10}

Inclusion and exclusion criteria can be found in Table 1.

**Table 1:** Inclusion and exclusion criteria

Inclusion criteria	Exclusion criteria
(a) Evidence of a stroke without involvement of the cerebellum at least 6 months prior to enrollment	(a) Orthopedic conditions of either the less affected or affected wrist
(b) FMUE score between 23 and 38	(b) An MMSE score lower than 21
(c) 0 through 3 in wrist flexion and wrist extension on the Modified Ashworth Scale	(c) *A stroke in the cerebellum*
(d) Individuals who are at least 18 years old and have the ability to consent.	(d) *History of epilepsy, seizures, or convulsions*
	(e) Ringing in ears
	(f) Presence of cochlear implant
	(g) Presence of pacemaker or neurostimulator
	(h) History of persistent headaches
	(i) Metal implant or fragments of metal in head or neck area
	(j) Presence of other neurological conditions including PD or CP
	(k) History of head trauma or concussion with loss of consciousness
	(l) Received Botox in the affected UE within the past 6 months
	(m) Metastatic cancer
	(n) Prisoners, children, or pregnant women
	(o) Individuals under the age of 18
	(p) Any adult unable to consent

Eligibility criteria for clinicians working as standardized treatment therapists for this trial include being a registered and licensed occupational therapist. Assessments are performed by licensed occupational therapists who are not treating the participants. Research staff trained in TMS, collect the TMS data. A TMS safety checklist is administered to ensure no contraindications to TMS.

### Who will take informed consent? {26a}

If a participant is found to be eligible and wants to be in the study, informed consent is obtained by a research team member during the in-person screen. A model consent form is attached as an Additional file (see Additional file 2). The research is conducted in compliance with state and federal laws, including the Health Insurance Portability and Accountability Act (HIPAA) which requires researchers to protect and maintain confidentiality of an individual’s health information. All subjects are asked to sign an “Authorization to Use and Disclose (Release) Health Information for a Research Study.” All research staff are trained in proper procedures for obtaining informed consent.

Study staff complete all initial phone screens, in-person screens, and consenting of participants. “SOP: Informed Consent Process for Research (HRP-090)” is followed while obtaining consent from each individual participant.

### Additional consent provisions for collection and use of participant data and biological specimens {26b}

If the participant agrees, we collect videos of the participant performing functional tasks pre- and post-intervention. Videos are used to train therapists in the study protocol as well as demonstrate functional improvement during academic and scholarly presentations. The choice to videotape is clearly stated on the consent form. The participant has the right not to be videotaped. This trial does not involve collecting biological specimens for storage.

## Interventions

### Explanation for the choice of comparators {6b}

We are using the same treatment protocol as our pilot study. However, we have changed the comparator group design. The intervention for the control group (CP) is subthreshold electrical stimulation. We apply transcutaneous electrical nerve stimulation (TENS) to the volar aspect of the paretic arm for 15 min. The current is an asymmetrical biphasic square impulse waveform with a frequency of 40 Hz and a pulse width of 250 μs. The intensity is initially adjusted so that the subjects perceive the stimulation. It is then reduced to 1 milliamp (sub-sensory threshold) and maintained for 15 min*.* Consistent with our pilot study, we expect improvement in both groups. However, we expect a larger magnitude of improvement in the BMP group that will be most evident at follow-up.

Both groups participate in a different priming intervention but the same TST protocol. The difference between the comparators is the priming intervention. The choice of the subthreshold e-stim control group was to control for participant’s expectations and attention such that any difference between the two groups is due to BMP.

### Intervention description {11a}

There are fifteen, 2-h sessions of treatment over 5 weeks (approximately three times per week) for a total of 30 h of BMP + TST in the experimental group. The control group receives the same duration and schedule (30 h of CP + TST) for treatment. A sixth week is reserved for any make-up sessions needed. There are two, 1-h treatment sessions per study visit. A minimum 30-min break separates each hour of training. During the first session, participants receive 15 min of priming (either BMP or CP) followed by 45 min of therapist-selected activities from the TST protocol. The second hour includes 15 min of priming plus 45 min of training on tasks selected from the Canadian Occupational Therapy Measure (COPM) [22].

#### Priming

**Fig. 1:**
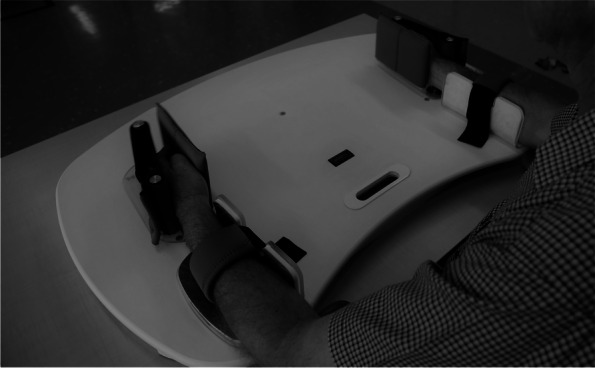
“Rocker” for bilateral priming. Legend: The Exsurgo priming device (Exsurgo Bilateral Primer, Auckland, New Zealand) used for bilateral priming in this protocol. Permission to use this image was granted by Exsurgo Rehabilitation Ltd. (see Additional file 3)

Priming precedes treatment in both intervention groups. During BMP, participants use the Exsurgo priming device (Exsurgo Bilateral Primer, Aukland, New Zealand) (see Fig. 1). For the participants assigned to the BMP group, both hands are strapped in place in the vertically oriented plates which are attached via a mechanical linkage. Both wrists move in rhythmic, symmetrical wrist flexion and extension for 15 min at a target frequency of 1 Hz as cued by a metronome. Participants do not need to have active flexion and extension of the affected hand because the less affected arm drives the movement of the paretic UE (through the mechanical linkage underneath the surface of the device). The device has a counter to keep track of repetitions with an ideal goal of 900 repetitions per 15 min of priming. The optimal *daily* (inclusive of both sessions) goal of priming repetitions is 1800. Participants are encouraged to strive to meet the goal.

#### TST treatment

The first session includes tasks from a TST protocol that has been used and shown efficacy in previous clinical trials [13, 23–26]. The TST protocol includes both unimanual activities and bimanual activities. Tasks are designed to improve components of upper extremity control such as transport, grasp, grip, release, and manipulation. The therapist selects 3–4 activities or exercises from the TST protocol based on the specific needs of the participant. The specific activities and number of repetitions are recorded. Emphasis is placed on increasing repetitions as client ability and task difficulty allow.

The second of the two daily sessions includes practice in two or more activities identified by the participants as both meaningful to them and needing improvement. The Canadian Occupational Performance Measure (COPM) [22] is administered at the baseline assessment appointment and is used to guide treatment. The activities of daily living (ADL) skills must have an UE component to them and can be unimanual (i.e., brushing hair) or bimanual (i.e., stirring a cake mix). Both Sainburg and colleagues [27] and Kantak et al [28] have stressed the importance of using bimanual asymmetric tasks in stroke rehabilitation. These tasks are often used during performance of ADL. During bimanual task training, the affected arm can either be used as a stabilizer (i.e., the arm holding the bowl) or as the dominant manipulator (i.e., the affected arm performing the mixing). The level of use of the affected arm (i.e., stabilizer or manipulator) is determined by goals of the participant, ability of the affected arm, demands of the task, and pre-morbid hand dominance. Activities are graded by the clinician to achieve a “just right” challenge, and emphasis is placed on increasing repetitions as participant ability and task difficulty allow. The specific tasks and the number of repetitions are recorded.

#### Home program

At the end of the first treatment day, therapists issue participants instructions for completing a set of active movements outside of therapy. Participants are given three sets of upper extremity movements targeting stretching, range of motion, and/or muscle strengthening. Targeted muscle groups depend on individual needs as determined by the therapist (i.e., wrist flexion, extension, and radial/ulnar deviation; forearm pronation and supination; elbow flexion and extension; digit extension, flexion, abduction, and adduction; shoulder flexion, extension, abduction, internal rotation, and external rotation). Therapists instruct participants to complete these specified movements 10–15 min daily. A handout with instructions is provided to ensure carryover.

#### Therapist training

All treatment therapists are trained in the administration of priming and TST protocols by the author of the protocol (MES). Subsequently, therapists must complete a formal standardization process that consists of the therapist(s) participating in a mock treatment session with a person with stroke. In order to pass the standardization, the therapists must score 90% on an itemized standardization checklist. Items on the checklist include the following: (1) grades activity to provide just right challenge; (2) able to downgrade activity when necessary; and (3) adapts environment to optimize performance. Therapists must pass a re-standardization test every 6 months. When needed, therapists meet with the investigator in charge of treatment fidelity to review videos and discuss treatment plans, treatment goals, and the progress of therapy.

### Criteria for discontinuing or modifying allocated interventions {11b}

Participation in this study can be terminated by the investigator without participant consent if circumstances arise that warrant doing so. This would include injury that would limit participation in treatment or testing or if the person becomes ill during the research study. A decision would be made to protect the health and safety of the participant. Participants may also voluntarily withdraw from the treatment at any time point. If continuing with evaluations is not harmful to the participant, we request that they complete post-evaluation and/or 8-week follow-up evaluation.

### Strategies to improve adherence to interventions {11c}

Adherence to the interventions has three components. This includes attendance to scheduled appointments, completing the assigned priming for 15 min per session, and completing a sufficient number of repetitions per session in the TST protocol. Although the ability to perform repetitions varies among participants, we expect that participants should reach a minimum of 100 repetitions of TST over 90 min of treatment. Treatment therapists continuously remind participants of this goal.

Regarding attendance, all participants receive a hard copy of their treatment schedule to promote adherence. If a participant is having difficulty keeping track of the sessions, reminder phone calls to the participant or the participant’s significant other or next of kin is initiated. We consider a participant 100% adherent for attendance if they complete the total of 30 h priming and training within 6 weeks.

### Relevant concomitant care permitted or prohibited during the trial {11d}

Participants are not permitted to be in occupational therapy treatment during the study and cannot be involved in any other physical rehabilitation studies.

### Provisions for post-trial care {30}

If participants require any care during or after their participation in this trial, the study team will follow the policies of the Shirley Ryan AblityLab and the Northwestern PTHMS Department. We do not expect greater than minimal harm from trial participation. If harm should arise, whether related to research protocol or not, we do not offer any compensation.

### Outcomes {12}

Each participant is evaluated with primary and secondary outcome measures, TMS measures, and additional outcome measures at pre-intervention, post-intervention, and 8-week follow-up.

#### Primary outcome measure: Fugl-Meyer Upper Extremity Test (FMUE)

The primary outcome measurement is the FMUE, an impairment scale with established interrater and intrarater reliability that addresses both synergy and isolated movements of the upper limb [12, 29, 30]. It comprises nine subscales that include reflex activity, dynamic movement within flexor synergy, dynamic movement within extensor synergy, movements mixing flexor and extensor synergies, movements out of synergy, normal reflex activity, wrist stability and mobility, hand, and coordination/speed subscales. The total score ranges from 0 to 66. The pre-intervention to follow-up change score (follow-up – pre) was selected because previous priming-plus-training studies have documented that the largest difference between groups occurs at follow-up [13, 16, 18].

#### Secondary outcome measure: Chedoke Arm & Hand Activity Index 9 (CAHAI-9)

The secondary outcome measure, the CAHAI-9, is a bimanual function test. It has strong support for its cross-sectional validity, test-retest reliability, and sensitivity to change [31, 32]. The CAHAI-9 was chosen because, in individuals with severe UE hemiparesis, use of the affected hand most often occurs in bimanual tasks*.* CAHAI-9 involves nine activities including opening a jar, pouring water, drawing a line with a ruler, buttoning a shirt, using the telephone, wringing out a wash cloth, applying toothpaste to a tooth brush, cutting food, and drying one’s back with a bath towel. The scale range is from 1 to 7 for each test item (each bilateral activity). The test items are graded by the amount of use of the affected hand. The affected hand can be used as a stabilizer or manipulative and is not graded down if used in a stabilizer role. The change score from pre-intervention to follow-up (follow-up–pre) will be analyzed as previous priming studies have documented the largest difference between groups occurs at follow-up [13].

#### Transcallosal inhibition

The primary TMS measure documenting possible neurophysiological change is transcallosal inhibition (TCI) from the ipsilesional to the contralesional hemisphere. TCI is collected via the ipsilateral silent period (iSP) using single-pulse TMS. Muscle activity is recorded from the extensor carpi radialis (ECR) of the affected and less affected forearms with surface EMG. Data is recorded for analysis using Signal 6. Magnetic stimuli are delivered using the MagStim 200 and a focal figure-of-eight coil (wing diameter 9 cm).

To begin, the participant is seated in a chair with their arms supported in a resting, pronated position. The optimal coil position for eliciting motor evoked potentials (MEPs) for the ECR is determined first for the contralesional hemisphere and subsequently for the ipsilesional hemisphere. Following hot spotting, the resting motor threshold (RMT) is obtained by increasing or decreasing stimulator output to find the minimal intensity at which 4 out of 8 resting MEPs with a peak-to-peak amplitude of 50 microvolts can be elicited.

For those without MEPs on the paretic side, coil location of the contralesional hemisphere is mirrored. If we cannot obtain resting MEPs from ipsilesional hemisphere, we attempt to elicit active MEPs (during active movement) using the criteria documented by Stinear et al. [33]. The elicitation of active MEPs determines the MEP status of participants who are subsequently documented as either MEP(+) or MEP(−). MEP status is a biomarker, and it is widely believed that individuals who are MEP+ have better chance of improvement. Documenting MEP status in a clinical trial is considered best practice [34].

During the experiment to elicit an iSP, both hands of the participant remain positioned in pronation, and participants are instructed to extend the wrist ipsilateral to the stimulated hemisphere and generate a voluntary contraction of 50% of the averaged maximum voluntary effort. Real-time, computerized visual feedback is provided to assist participants in the accuracy of maintaining effort at 50%. Meanwhile, 16 stimuli (one stimulation every 5 s or .2 Hz) are delivered over the ECR hotspot at 150% RMT or 80% maximum stimulator output (MS), whichever is greatest. If RMT in the ipsilesional hemisphere cannot be obtained, the participant receives stimulation at 100% MSO. Rest breaks of approximately 30 s are given every 4 stimuli to prevent fatigue. The stimulation procedure is repeated for the contralesional hemisphere.

#### Additional outcome measures: Action Research Arm Test (ARAT)

The ARAT is a test of unilateral function and includes grasp, grip, pinch, and whole arm movement subscales [35]. Total scores range from 0 to 57. The ARAT is often used in post-stroke rehabilitation studies as a primary outcome measure [36–38] or secondary outcome measure [39]. As such, we have included it as an additional outcome measure. We predict that if changes are observed, they will be more evident in our higher strata (individuals with FMUE scores between 30 and 38) at the follow-up time point.

#### Grip termination time (GTT) and maximal grip strength

The GTT measures the time it takes for an individual to release an object and is administered following the methodology previously published [40]. Both the affected and less affected hand are evaluated. Electrodes are placed over the flexor digitorum superficialis (FDS) as well as extensor digitorum communis (EDC) of both forearms following standard skin preparation techniques. Maximal voluntary activation of each muscle as well as muscle activation onset time and offset time, indicated to the participant by an auditory cue, is recorded. Maximal grip strength is also documented from a hand dynamometer.

#### Neuro-QoL

The Neuro-QoL is a self-report measure that assesses patient experience of neurological conditions and treatment for such conditions through quality-of-life domains including mental, social, and physical health [41]. The instrument has high reliability and established internal consistency and is self-administered via Research Electronic Data Capture (REDCap) survey. Neuro-QOL is a Common Data Element (CDE) measures, which are population-specific measures either strongly recommended or required. CDE measures enable clinical investigators to systematically collect, analyze, and share data across the research community.

### Participant timeline {13}

**Fig. 2:**
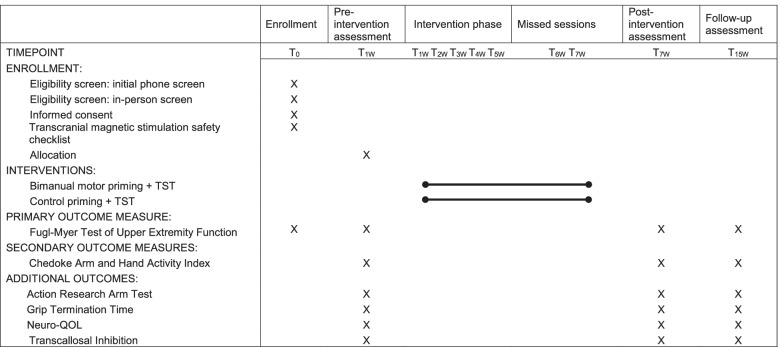
Schedule of enrollment, interventions, and assessments. Legend: Participants who are eligible are enrolled, participate in baseline assessments, are allocated to treatment group, and receive 5 weeks of intervention with the 6th week used to make up for any missed sessions. The post-treatment assessments occur between weeks 6 and 7 and follow-up assessments occur between weeks 13 and 15.

**Fig. 3:**
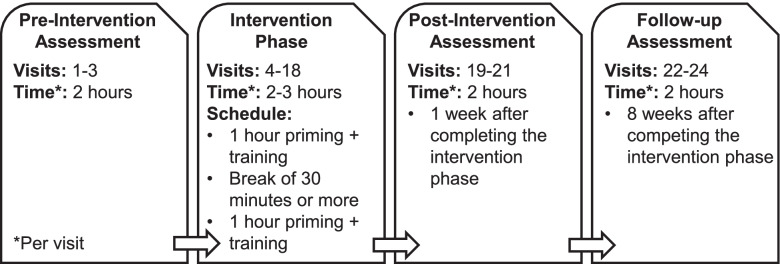
Schedule of visits and time per visit. Legend: Participants complete up to 24 visits over 15 weeks, with sessions lasting from 2 to 3 h each. The schedule for visits during the intervention phase will consist of 1 h of priming + task-specific training, then a break of at least 30 min, followed by another 1 h of priming + task-specific training

The duration of an individual’s participation in the study is approximately 4–6 months. The schedule of enrollment, assessment, and intervention is depicted in Fig. 2. Figure 3 presents an outline of an individual’s visit schedule including the type of visit (evaluation or treatment) and the number of hours per session. Once the individual completes baseline evaluations, treatment begins in the same or following week. We allow 6 weeks for treatment (5 weeks with an additional week for missed sessions). The primary investigator determines if an extension for treatment beyond 6 weeks is allowed for a particular participant.

### Sample size {14}

We are enrolling 38 subjects per group (*N* = 76). Allowing for the attrition of 12 participants (15% based on our pilot study [13]), we project at least 32 subjects per group will have both the baseline and follow-up data required to compute our change outcomes at 8 weeks post-treatment cessation. For the hypotheses tested for objectives 1 and 2, we will evaluate between-group (BMP+TST vs. CP+TST) differences using analyses of covariance (ANCOVA) that adjust for the outcome measure at baseline; tests will be two-sided at the 5% level (*α* = 0.05). The power of these tests depends on the number of participants in each group, the separation of the means of each group, the significance level, the population standard deviation (SD) of the outcome measures, and the correlation between measures at baseline and follow-up. We consider what sample size per group (*n*) will provide 90% or greater power.

We discuss each of the two objectives in turn. Table 2 presents the primary outcome measure (FMUE), secondary outcome measure (CAHAI-9), and the additional outcome measure (TCI in objective 2). For each measure, the table shows the difference in the group means that can be detected with 90% power assuming *N*=32 per group, along with the hypothesized SD of the outcome. Power computations assume a modest correlation between baseline and follow-up for measures of *R*^2^ = 0.2. To derive our estimates of the SD of outcome measures at follow-up (8 weeks post-treatment cessation), we used the results of a published pilot study at 6 weeks post-treatment cessation [13].

**Table 2:** Sample size analysis: primary and secondary measures

	Mean difference	SD
**Aim 1**		
**FMUE**	5.98	8.11
**CAHAI-9**	5.34	7.26
**Aim 2**		
**TCI**	2.74	3.71

Effects detectable with 90% power for Aims 1 and 2

*Abbreviations*: *SD* standard deviation of the change score (follow-up–pre), *FMUE* Fugl-Meyer Upper Extremity Function, *CAHAI-9* Chedoke Arm & Hand Activity Index-Nine, *TCI* transcallosal inhibition

### Recruitment {22}

Participants are recruited from the Northwestern University and Shirley Ryan AbilityLab Clinical Research Registry, a registry that identifies individuals who consent to be contacted for research purposes post-stroke. The Clinical Research Registry began in 2001 and has compiled the information of over 776 individuals post-stroke, including names, contact information, and clinical characteristics (e.g., side of lesion, date of stroke, level of arm impairment).

Other recruitment methods include reaching out to stroke support groups in the Chicago area connected with neurologists in nearby medical centers (i.e., Northwestern University, University of Illinois, and University of Chicago). We also post IRB-approved flyers in the community. Referrals are accepted through doctors and therapists. Potential participants can also contact the investigators independently.

## Assignment of interventions: allocation

### Sequence generation {16a}

Prior to randomization, participants are stratified according to the FMUE measured at baseline. Participants with a FMUE score of 23 through 29 are stratified to the severely impaired group, and those with scores of 30 through 38 are stratified into the moderately impaired group. Each impairment stratum has its own computer-generated random number list. Since participants are stratified, we plan to conduct exploratory analyses on the extent to which the severity of impairment alters treatment outcome.

### Concealment mechanism {16b}

To ensure concealment, a research assistant records randomization assignment in a password-protected document and communicates the participant’s priming assignment via data recording sheets kept in a locked cabinet inaccessible to masked individuals.

### Implementation {16c}

Once the participant completes baseline evaluations, FMUE scores are communicated to the study coordinator who assists with allocation. The study coordinator contacts a specific member of the investigative team with the following information: (1) FMUE category (severe or moderate) and (2) number of the randomized participant. This investigator holds the randomized computer-generated lists for both stratification levels and allocates group assignment. The investigator has limited contact with study participants and is not involved in the day-to-day aspects of the treatment or evaluation. The study coordinator then communicates group assignment to the occupational therapists on the team.

## Assignment of interventions: masking

### Who will be masked {17a}

Members of the investigative team including therapists administering motor assessments and individuals collecting, processing, and analyzing the TMS data are masked to treatment allocation. Treatment therapists and the participants are not masked for practical reasons. All evaluations occur in a different space than treatment. Participants are reminded not to discuss group assignment or treatment with any individual administering assessments.

### Procedure for unmasking if needed {17b}

The only circumstance under which unmasking is permissible is in the case of a participant experiencing a serious adverse event. In this case, only the medical safety monitor for the trial is informed of the allocation to intervention during the trial. The unmasking procedure will not include any study staff member or member of the investigative team who is masked.

## Data collection and management

### Plans for assessment and collection of outcomes {18a}

Behavioral assessments, TMS measures, and grip termination time (GTT) are collected for all participants at baseline, post-treatment evaluation, and an 8-week follow-up evaluation. All raters must pass a standardization test with a score of 95% to insure proper assessment administration. Procedures are followed according to published directions [12]. Subsequent standardizations occur every 6 months. Staff members administering GTT and TMS are thoroughly trained in all procedures.

### Plans to promote participant retention and complete follow-up {18b}

If a participant misses scheduled appointments more than one time, the therapist(s) speaks to the participant about the necessity of showing up for appointments and participating in the intervention. If this does not produce a change in behavior, the investigator then speaks with the participant and emphasizes the importance of consistent attendance. Individuals who have difficulty remembering appointments receive regular phone calls. They are also contacted prior to the 8-week follow-up appointment.

### Data management {19}

Completed de-identified data sheets as well as consents with protected health information are signed by the study staff member, and the recorded data is scanned to a web-based data system that is password protected. Data is backed up on the web-based system as well as computer hard drives. Data from the behavioral assessments are entered into REDCap by selected study staff. TMS data is collected and stored on a primary lab computer hard drive and backed up on an external hard drive at regular intervals.

The first level of monitoring is carried out by the study coordinator and includes checking all informed consents, evaluations, and treatment records. All errors are reviewed, and the coordinator verifies that errors are crossed out and annotated with the researcher’s signature and date in the hard copy of data sheets. The study may also be monitored by a pre-arranged visit from a representative of the sponsor’s IRB office. Specific participant files that may be in an audit include consent forms, consent process forms, and evaluation forms. Delegation of authority logs and other items from the regulatory binder are also examined.

### Confidentiality {27}

Hard copies of screens containing demographic and protected health information are stored in locked files accessible to the study coordinator. Hard copies are also scanned into a server protected by a firewall from Northwestern University. Selected study members have access to the server. De-identified demographics are stored in REDCap. A limited number of study personnel (including study staff that perform screenings or are involved in the consent process) have access to REDCap. De-identified data will be shared with the sponsoring agency if requested. The investigative team is responsible for receipt and transmission of the data.

No information about participants or provided by the participants during the research will be disclosed to others without their written permission, except (1) if necessary to protect participant’s rights or welfare (for example, if they are injured and need emergency care or when the Institutional Review Board monitors the research or consent process); or (2) if required by law.

### Plans for collection, laboratory evaluation**,** and storage of biological specimens for genetic or molecular analysis in this trial/future use {33}

See above 26b, there will be no biological specimens collected.

## Statistical methods

### Statistical methods for primary and secondary outcomes {20a}

Data will be stored in SAS data files and statistical analyses will be conducted using SAS. We will use an intent-to-treat analysis for our primary and secondary analyses. At baseline, post-intervention, and 8-week follow-up time points, standard descriptive statistics will be calculated for degree of impairment (FMUE) and degree of function (CAHAI-9). We will also compute standard descriptive statistics for less severely impaired participants (FMUE 30-38) and the more severely impaired participants (FMUE 23-29) as determined at baseline. We will assess the relationship between both FMUE and CAHAI-9 at baseline and time since stroke, age, and gender. In addition, we will examine the correlation between changes in FMUE and CAHAI-9 from baseline to follow-up time points (end of intervention and 8 weeks post-treatment cessation) and time since stroke, age, and gender. We will report pairwise correlations and standard errors for these descriptive analyses.

Confirmatory analyses will largely use normal theory methods based on analysis of covariance. We will use residual diagnostics to evaluate model fit and take appropriate data transformations as necessary.

#### Hypothesis 1a and 1b analysis

For each dependent measure (FMUE and CAHAI-9), primary confirmatory analyses will compare the experimental group (BMP+TST) versus the control group (CP+TST). We will use normal linear models with effects for treatment assignment and the outcome measure at baseline, equivalent to an analysis of covariance. We will report point estimates and standard errors of these differences and conduct two-sided null hypothesis tests at the 5% level (*α* = 0.05).

#### Hypothesis 2a and 2b analysis

Descriptive statistics for Hypotheses 2a and 2b will follow a similar approach as those for 1a and 1b, but we will focus on TCI as our outcome of interest. Confirmatory analyses will analyze differences in TCI between treatment arms at 8 weeks post-treatment cessation. We will report estimated differences between treatment arms along with standard errors. We will also conduct two-sided hypothesis tests at the 5% level. For 2b, we will perform a correlation to determine the relationship between changes in ipsilesional TCI and FMUE from pre to follow-up.

### Interim analyses {21b}

Not applicable. At this time, we have no plans for an interim analysis.

### Methods for additional analyses (e.g., subgroup analyses) {20b}

As exploratory analyses, we will use linear regression models outcomes at 8 weeks post-treatment cessation as the dependent variable (FMUE, CAHAI-9, and TCI). These models will include treatment assignment (BMP vs. CP) and baseline outcome measure as a fixed-effects, as well as patient characteristics (age, low or high impairment severity at baseline, sex, and MEP status). We will examine differential impacts by fitting separate models with treatment-impairment severity, treatment-sex, and treatment-MEP status interactions. We will report relevant point estimates and standard errors for these models and conduct 5% level tests for interaction terms with two-sided alternative hypotheses.

### Methods in analysis to handle protocol non-adherence and any statistical methods to handle missing data {20c}

We will measure protocol adherence by the median number of repetitions of TST repetitions. Treatment therapists record the number of repetitions for each activity, and the data is de-identified and transferred to an excel sheet. Participants will be labeled as adherent if the median number of daily TST repetitions (over the 2-h visit) is equal to or greater than 100. Information about treatment activities is only available to non-blinded individuals.

Though we plan to follow up with patients repeatedly to minimize the amount of missing data, we expect some data may still wind up missing. Should greater than 5% of data be missing, we will explore missingness patterns via graphical analyses to evaluate potential mechanisms of missingness. If appropriate, multiple imputation will be used to handle missing data.

### Plans to give access to the full protocol, participant-level data, and statistical code {31c}

Access to the full protocol and participant-level data will be considered upon submission of a reasonable request.

## Oversight and monitoring

### Composition of the coordinating center and trial steering committee {5d}

The implementation of the trial is overseen and monitored by the trial steering committee. The trial steering committee is composed of the principal investigator [DMC] who is ultimately responsible for the trial, the co-investigator [MES], study coordinator [AW], and the experimentalist [EK]. All members of this committee are responsible for recruitment and retention activities. Select research staff are involved in the consent process and must be identified as such in the IRB study protocol.

### Composition of the data monitoring committee, its role and reporting structure {21a}

We have established a data safety and monitoring board (DSMB) that consists of international experts in therapeutic interventions as well as TMS with the post-stroke population. They provide a mechanism to assure monitoring of the overall conduct of the study (including issues with safety, ethics, patient recruitment and accrual, retention, and adequacy of study design to achieve the specific aims). They provide feedback to the study team for the possible protocol amendments. The DSMB also reviews serious adverse events (SAEs) and adverse events (AEs) and are alerted to any interim concerns. The DSMB is independent of the study sponsor and have no competing interests.

### Adverse event reporting and harms {22}

Safety events are reported by the participant or observed by the research staff. Events are relayed to the PI and the safety monitor to determine next steps and if there is a possible relationship to the event and the research protocol. All events are documented and stored in a specific file on the firewall-protected server. The safety monitor, an MD, determines whether the event is categorized as an AE or an SAE, and oversees any notifications to the Northwestern University IRB. Review of all safety data occurs at every DSMB meeting. The DSMB report is signed by the PI and the Safety Monitor. It is filed with the IRB during annual continuing review. Data reviewed includes SAEs, AEs, and unusual changes in behavioral and TMS measures.

### Frequency and plans for auditing trial conduct {23}

Northwestern University IRB monitors the conduct of all clinical trials. The Northwestern IRB conducted a post-approval monitoring visit during the second year of the trial. The study team prepared items using a checklist provided by the IRB including a Post Approval Monitoring Checklist, Clinical Trial Checklist, and the Participant File Checklists for a selected number of participants. On-line and hard copies of participant files are reviewed at monitoring visits. The IRB notified the study team of minor findings in the study documentation that needed attention (i.e., form completion). The study team replied to the report and documented how the findings would be addressed going forward.

### Plans for communicating important protocol amendments to relevant parties (e.g., trial participants, ethical committees) {25}

Protocol amendments are communicated to and approved by the Northwestern University IRB. All study staff receive notice of changes deemed appropriate by study coordinator.

### Dissemination plans {31a}

The investigative team plans to publish trial results in peer-reviewed journals emphasizing neurorehabilitation. We will also present the trial results at neuroscience and rehabilitation science conferences.

## Discussion

This is the first large-scale, randomized clinical trial to evaluate the effect of bilateral priming and a systematic task-specific training protocol in moderate to severe chronic upper limb hemiparesis. The effect of training is rigorously controlled by using the same TST protocol in both experimental and control groups. The control protocol is dose matched in time with the experimental bilateral priming and is provided to satisfy participant attention and expectations.

This trial addresses a need in the field of neurorehabilitation to find effective, easily accessible treatments for participants post-stroke who have lower prospects for motor recovery due to moderate to severe UE impairment. These patients make up 75% of all stroke survivors who currently have no access to interventions likely to result in clinically significant improvements in motor function. For example, participants with chronic UE impairment made only modest improvements in FMUE scores after completing intervention programs using different variations of robotic therapy [4, 42]. Mirror therapy has been used in post-stroke participants with a range of impairment levels [43, 44]. However, non-response to mirror therapy is frequent and is related to lesion location [45] and initial impairment level [46]. In contrast, our previous findings and the findings of others indicate that bilateral priming may provide consistent clinically significant improvements in post-stroke hemiparesis [11, 13, 18].

Enrollment for this trial is ongoing at the time of publication. Analysis will occur when all participants complete the trial. Consistent with our previous studies, we expect an improvement in arm function in both the experimental and control group. We expect differences in improvement to be most evident at the follow-up time point. A positive outcome of this trial will emphasize the role of bilateral priming in rehabilitative training. Based on previous results of priming with rehabilitation protocols, such as video game-based movement therapy [11] or home programs emphasizing fine motor control [18], we expect that BMP will be applicable to many forms of post-stroke motor training other than task-specific therapy.

We expect the BUMP trial to demonstrate the additive effect of bilateral priming on our task-specific training therapy. However, we are aware that previous large trials for upper limb hemiparesis post-stroke have failed to detect significant between-group differences [4, 47, 48] and, in some cases, pre-/post-intervention effects have been small [36]. We expect the magnitude of change in the BMP+TST group to be greater than the change in the CP+TST. We may also demonstrate a large pre-/post-improvement on a more chronic post-stroke population. As previously stated, Ward and colleagues demonstrated a large improvement in the FMUE following 90 h of various types of upper limb therapy techniques [3]. We expect our improvement to be comparable to the Ward study. However, we expect similar improvements with only 30 h of intervention, one third of the dosage time used in the study by Ward and colleagues [3].

### Trial status

At the time of this publication submittal, we are recruiting participants with projected completion in June of 2023. This is protocol version 14. Recruitment began February 1, 2017.

## Supplementary Information

**Additional file 1.** Notice of award. Notice of award from the Eunice Kennedy Shriver National Institute of Child Health & Human Development.

**Additional file 2.** Model consent form. Model consent form for permission to take part in a human research study.

**Additional file 3.** Original ethics approval document. Approval of new study from the institutional review board office of Northwestern University.

**Additional file 4.** Model release. Model release form from Exsurgo Rehabilitation, Ltd., singed by the photographer R. Little.

## Data Availability

Any data required to support the protocol can be supplied on request.

## Abbreviations

ADLs: Activities of daily living
AE: Adverse event
ARAT: Action Research Arm Test
BMP: Bilateral motor priming
CAHAI: Chedoke arm and hand inventory
CDEs: Common Data Elements
CIMT: Constraint-induced movement therapy
COPM: Canadian Occupational Performance Measure
CP: Control priming
DSMB: Data safety and monitoring board
ECR: Extensor carpi radialis
FDS: Flexor digitorum superficialis
FMUE: Fugl-Meyer Upper Extremity Function
GTT: Grip termination time
IRB: Institutional review board
iSP: Ipsilateral silent period
MD: Licensed medical doctor
MEPs: Motor evoked potentials
MMSE: Mini-Mental Status Exam
MSO: Maximal stimulator output
MVC: Maximal voluntary contraction
Neuro-QoL: Quality of life in neurological disorders
PI: Principal investigator
PTHMS: Physical therapy and human movement science
rTMS: Repetitive transcranial magnetic stimulation
RMT: Resting motor threshold
SAE: Serious adverse event
SD: Standard deviation
TCI: Transcallosal inhibition
tDCS: Transcranial direct current stimulation
TMS: Transcranial magnetic stimulation
TST: Task-specific training
UE: Upper extremity
