# Supplementary material for: Bilateral upper extremity motor priming (BUMP) plus task-specific training for severe, chronic upper limb hemiparesis: study protocol for a randomized clinical trial
Source: Trials. 2022 Jun 22;23:523. doi: 10.1186/s13063-022-06465-9 (PMC9214193; doi:10.1186/s13063-022-06465-9)
Supplement: Supplementary file 2 — Additional file 2. Model consent form. Model consent form for permission to take part in a human research study. [file 13063_2022_6465_MOESM2_ESM.pdf]

Permission to Take Part in a Human Research Study

Do not sign this consent if today's date is later than the stated expiration date above.

Title of Research Study: Bilateral Priming plus Task Specific Training for Severe Upper Limb Hemiparesis, v. 10

Version 10: 05/22/2020

Investigator: Daniel M. Corcos, PhD

Supported by: This research is supported by National Institutes of Health.

Key Information:
The first few pages of this document include a summary of this study to help you decide whether or not to participate. Detailed information is provided after the summary.

Why am I being asked to take part in this research study?
We are asking you to take part in this study because you have had a stroke leaving you with a weak arm, and you have no history of seizures. .

What should I know about a research study?

- Someone will explain this research study to you.
- Whether or not you take part is up to you.
- You can choose not to take part.
- You can agree to take part and later change your mind.
- Your decision will not be held against you.
- You can ask all the questions you want before you decide.

Why is this research being done?
The purpose of the study is to determine the best treatment for the arm that has been affected by a stroke. It has been difficult for researchers to determine the optimal treatment for the weaker arm after a stroke. We plan to determine if your arm will improve with a combination of motor priming and motor training. Motor priming provides a warm up for the brain so that it will better respond to treatment. There are two types of priming in this study. One is called bilateral motor priming which involves using both hands. Bilateral priming requires that the individual make continuous wrist movements in a low-tech gadget called the Exsurgo primer. The Exsurgo primer is a piece of equipment in which each hand goes between two plates that are connected together so that the stronger wrist moves the weaker wrist in and out at the same time. Please see picture below (Figure 1). The Exsurgo primer has been used in several inpatient stroke research studies. It is also used in rehabilitation hospitals. The second type of priming includes use of low intensity stimulation for your affected arm. Understanding the benefits of priming combined with treatment might improve intervention for future patients with stroke.

Permission to Take Part in a Human Research Study
Do not sign this consent if today's date is later than the stated expiration date above.

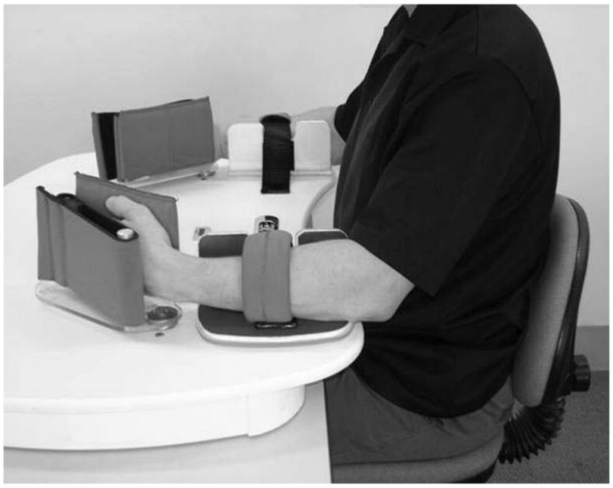

Figure 1. Exsurgo Bilateral Priming Device

How long will the research last and what will I need to do?
We expect that you will be in this research study for up to 4 to 6 months depending on how soon you are scheduled after you are enrolled.

After it has been decided that you can participate in this study, you will be asked to attend baseline testing which will include three sessions. After baseline testing (3 sessions), you will have 15 treatment sessions lasting approximately 3 hours (with an hour break between the first and second treatment hours). This will occur over 5-7 weeks. You will then have post-treatment testing (3 sessions). Eight weeks after your last day of treatment, you will have a follow-up. More detailed information about the study procedures can be found under the section **What happens if I say "Yes, I want to be in this research"?**

Is there any way being in this study could be bad for me?
This study uses TMS for testing brain function. Rare cases have reported the development of seizures during or immediately after magnetic brain stimulation. Individuals who have a history of seizures or have been diagnosed with epilepsy will be excluded from this research study. If you have a shunt, pacemaker or cochlear implants, you will not be eligible for the study due to possible heating from the coils that could compromise your safety.

More detailed information about the risks of this study can be found under **"Is there any way being in this study could be bad for me? (Detailed Risks)"**

Will being in this study help me anyway? We cannot promise any research to you or others from your taking part in this research. However, possible benefits include improved strength, motion and function in your arm and hand as well as improvement in the way that you perform specific self-selected tasks. Depending on the particular tasks that you select, improvement could improve your enjoyment of performing those tasks as well as benefit others involved in

those specific tasks. For example, practicing childcare tasks could benefit you as well as the child.

What happens if I do not want to be in this research?

Participation in research is voluntary. You decide whether to participate. If you choose to not participate, there will be no penalty to you or loss of benefit to which you are entitled. Instead of being in this research study, your choices may include: if you have insurance, you can obtain a referral for conventional outpatient occupational therapy from your doctor or you may choose not to be in study.

Detailed Information:

The rest of this document includes detailed information about this study (in addition to the information listed above).

Whom can I talk to?
If you have questions, concerns, or complaints, or think the research has hurt you, talk to the research team at Northwestern University. You may speak with Dr. Mary Ellen Stoykov, principal investigator of the Shirley Ryan AbilityLab team or the rincipal Investigator of research team (312) 503-3106 or Dr. Daniel M. Corcos (email: daniel.corcos@northwestern.edu; telephone: (312) 908-6792; cell: (708) 214-5454; or fax: (312) 908-0741.

This research has been reviewed and approved by an Institutional Review Board (IRB). You may talk to them at (312) 503-9338 or irb@northwestern.edu if:

- Your questions, concerns, or complaints are not being answered by the research team.
- You cannot reach the research team.
- You want to talk to someone besides the research team.
- You have questions about your rights as a research participant.
- You want to get information or provide input about this research

How many people will be studied?
We expect about 76 individuals with stroke from the Chicago or surrounding area will be in this research study.

What happens if I say "Yes, I want to be in this research?
You will have 24 visits. Some visits will be for evaluation and some for therapy. Each visit will be two to three hours depending on the type of visit and tests being done. Baseline motor evaluation, transcranial magnetic stimulation (TMS) and evaluation of grip termination will last approximately 2 hours. Each therapy visit will last approximately 3 hours (2 hours of training plus time for a break). If for some reason the equipment does not work during one of your regularly scheduled visits, we may ask you to come back on a different day, which would then

result in an additional study visit beyond the 24 regularly scheduled ones. Please see Figure 2 for a timeline of these study visits.

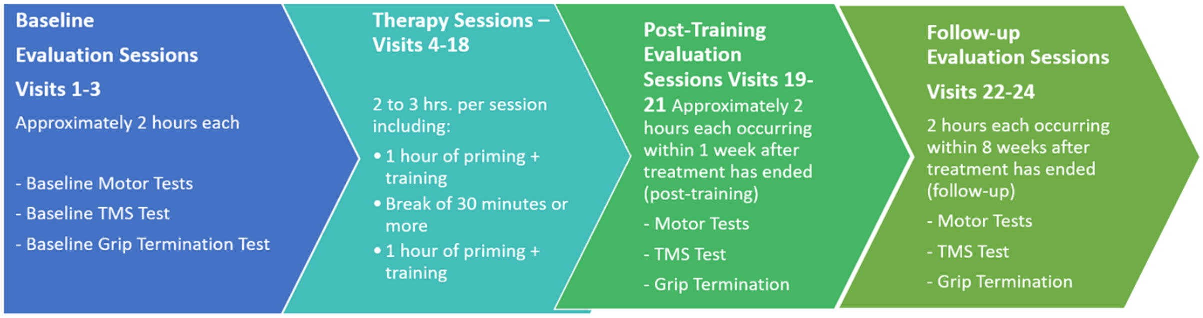

Figure 2. Timeline of Study Visits

All evaluation visits will take place at 645 North Michigan Avenue, Department of Physical Therapy, 11th Floor, Chicago IL, 60611. The Grip Termination Test will be completed at either Shirley Ryan Ability Lab or Northwestern University.

Evaluation sessions consist of three separate days of testing and will occur at three time points: (1) before treatment starts (baseline evaluation: visits 1-3); (2) after treatment is completed (post training evaluation: visits 19-21); and (3) 8 weeks later (follow-up evaluation: visits 22-24).

All treatment sessions will occur at Shirley Ryan AbilityLab at 355 East Erie, Chicago, IL 60611

Below is the detailed schedule of visits:

Visit 1: Baseline Motor assessments (approximately 2 hours). You will be tested on the Fugl Meyer Test of Motor function (FMUE). It is a common test for movement of the arm after you have had a stroke. We will also test you on the Chedoke Arm and Hand Activity Index (CAHAI). This test uses both hands and it tests your ability to do two-handed tasks like buttoning. You will also complete Action Research Arm Test that measures your ability to manipulate objects with your weaker arm only. Finally, you will complete the Neuro-QOL (short form). One of the subtests is the Depression Inventory that will ask questions about your emotional state. . If you exhibit depressive symptoms at any time, we will monitor the Depression Inventory and take appropriate actions if you indicate a high risk of suicide. However, this question is optional and you may opt out of answering this question and continue participation in the research study.

Visit 2: TMS Evaluation (approximately 2 hours). After a stroke, there is often a change in the balance of excitability between the affected and less affected p of the brain. We will measure this balance using Transcranial Magnetic Stimulation (TMS), a technique used in neurorehabilitation research. <u>We will not use TMS for treatment.</u> Recording devices called electrodes will be taped to the skin on your wrist using adhesive pads. Wires from the electrodes will be connected to a computer for recording your muscle activity and responses from the TMS. TMS involves sitting in a chair while an insulated coil is positioned on top of your

head delivers magnetic pulses. The coil makes a clicking sound and will produce a twitch in your muscles. The magnetic pulse induces a weak electrical current in your brain, in the same way that you produce a weak electric current in your brain when you voluntarily make a muscle contract. You will be asked to contract your wrist muscle, and we will apply TMS stimulation to allow us to test inhibition levels in your brain. Inhibition refers to prevention of movement by the brain. It is a normal function of the brain. However, after stroke, there may be too much inhibition. You will be able to rest at any time during a session, postpone the remainder of a session, or you may withdraw from the study at any time. You will not undergo TMS during the training.

Visit 3: Grip termination evaluation (approximately 1-2 hours). This test will determine how long it takes to relax your hand after gripping an object. Electrodes will be taped on your arm. We will ask you to squeeze an object as hard as you can. When you hear an auditory tone, you will release the object. Electrodes (which are for recording purposes only) will measure the muscle activity.

Visits 4-18 **at Shirley Ryan AbilityLab**: Therapy Sessions (2 hours, plus additional time for a break). These visits will occur shortly after your first three visits are complete. You will have 3 visits a week (Monday thru Friday) over a period, for a total of 15 visits. You will have the chance to make up any missed sessions in week 6. If you are in the bilateral priming group you will make continuous wrist movements with a device that will assist your wrist(s) to flex and extend continuously for approximately 15 minutes. If you are in the electrical stimulation-priming group you will have some wrist stimulation. After the priming, motor training will be done with your affected arm for approximately 45 minutes. You will then take a break, which will be followed by another session of priming and training. We may increase the time for your break depending on your fatigue level.

Visit 19: Post-training motor evaluation (2 hours). This will occur within 10 days of completing all of your therapy sessions and will repeat the tests done at Visit 1.

Visit 20: Post-training TMS evaluation (2 hours). This visit will occur within ten days of completing all of your therapy sessions and will repeat the tests done at Visit 2.

Visit 21: Post-training Grip Termination evaluation (1-2 hours). This visit will occur within ten days of completing all of your therapy sessions (Visit 18) and will repeat the test done at Visit 3.

Visit 22: Follow up motor evaluation (2 hours). This visit will occur eight weeks after completing all of your therapy sessions and will repeat the tests done during visits 1 and 19.

Visit 23: Follow up TMS evaluation (2 hours). This visit will occur within 10 days of completing visit 22 and will repeat the tests done at Visits 2 and 20.

Visit 24: Follow up Grip Termination evaluation (1-2 hours). This visit will occur within 10 days of visit 22 and will repeat the tests done at Visits 3 and 21.

Permission to Take Part in a Human Research Study
Do not sign this consent if today's date is later than the stated expiration date above.

We will have a follow-up survey via phone or as part of an aforementioned follow up evaluation. The purpose of the survey is to learn about your motivation, confidence, and belief in your upper limb recovery. We will be asking about your experience since sustaining a stroke. You have the right to refuse the survey.

All procedures, including the occupational therapy, are part of the research study and not part of ongoing clinical care.

You may be contacted for future research.
You will be asked to complete a video of performing specific tasks. This is optional

The treatment you get will be chosen by chance, like flipping a coin. Neither you nor the study doctor will choose what treatment you get. You will have an ***equal*** chance of being given either treatment. You will know which treatment you are getting but your study doctor and evaluators will not know.

Detailed Risks: Is there any way being in this study could be bad for me?

Physical Risks

1.There are no serious risks to motor training and tests of motor function.. You may however, feel fatigue in your arm especially in the beginning of training. There is a slight chance that you may experience pain associated with muscle soreness after treatment or after motor or TMS testing. If soreness does not resolve, you should notify your occupational therapist. If the pain is intense and increases during treatment, you will be removed from the study.

2. There may be a risk of mild skin irritation at the location where the adhesive electrode sensors will be placed, but this usually consists of minor redness that will go away within a few hours after they are removed.3. Noise: During TMS, a loud click during magnetic stimulation may be heard. If this causes discomfort, you will be provided with foam earplugs to reduce or prevent the discomfort.

3. Headache: A mild headache can occur following TMS treatment that usually resolves soon after the procedure. The headaches may be from maintaining a fixed head position for the duration of the TMS testing. We will try to reduce the risk of headache by assuring your comfort before and during the procedures.

4. TMS Muscle Twitching: You will feel twitches in your muscles because of the magnetic stimulation of your brain. If this causes excessive discomfort, please let the researcher know. You can take rest breaks or chose to withdraw from the study at any time.

Psychological Risks:

You may feel frustrated if the tasks are difficult to perform.

Confidentiality Risk

This study involves the use of your identifiable, personal information and there is a chance that a loss of confidentiality could occur. The researchers have procedures in place to lessen the possibility of this happening. See the section below titled: **"What happens to the information collected for the research?".**

What do I need to know about reproductive health and/or sexual activity if I am in this study?

The research may also hurt a pregnancy or fetus in ways that are unknown. The risks of TMS to pregnant woman, fetuses, and nursing mothers are not known. These may be a minor inconvenience or may be so severe as to cause death. Women who are pregnant or are breast-feeding cannot take part in this study. You should not become pregnant or breastfeed while in this research study. There are no known risks to breast feeding mothers, and there is no reason to believe that TMS would have any effect on breast milk. However, this precautionary measure should be taken. After your last (follow-up) TMS session, you can discontinue birth control or resume breastfeeding an infant.

If you are female and sexually active, you should use at least one effective means of birth control while participating in this research study. According to the World Health Organization and the United States Center for Disease Control and Prevention, the most effective forms of birth control include complete abstinence, surgical sterilization (both male and female), intrauterine devices (IUDs), and the contraceptive implant. The next most effective forms of birth control include injectables, oral contraceptive pills, the contraceptive ring, or the contraceptive patch. Acceptable but least effective methods of birth control include male condoms (with or without spermicide) and female condoms. You should not be or become pregnant while on this research study. If you become pregnant while participating in the study, it is important that you tell the study doctor or another research team member immediately. You may be required to stop participation in this study.

If you are a postmenopausal woman, you are not required to use contraception while participating in this research study. Postmenopausal women rarely become pregnant. If you become pregnant while participating in this research study, it is important that you tell the study doctor or other research team member immediately. You may be required to stop participation in this study; however, other treatment options will be discussed with you at that time if necessary.

Will it cost me anything to participate in this research study?
Taking part in this research study will not lead to any costs to you.

Permission to Take Part in a Human Research Study
Do not sign this consent if today's date is later than the stated expiration date above.

What happens to the information collected for the research?
Efforts will be made to limit the use and disclosure of your personal information, including research study and medical records, to people who have a need to review this information. We cannot promise complete secrecy. Organizations that may inspect and copy your information include the IRB and other representatives of Northwestern or Shirley Ryan Ability Lab.

We will not ask you about child [or elder] abuse, but if you tell us about child [or elder] abuse or neglect, we may be required or permitted by law or policy to report to authorities.

The sponsor, monitors, auditors, the IRB, the Northwestern University Office for Research Integrity, the US Office of Research Integrity (ORI), the US Office for the Protection of Human Research Protections (OHRP), and the National Institutes of Health may be granted direct access to your medical records to conduct and oversee the research. By signing this document, you are authorizing this access. We may publish the results of this research. However, we will keep your name and other identifying information confidential.

Data Sharing
De-identified data from this study may be shared with the research community at large to advance science and health. We will remove or code any personal information that could identify you before files are shared with other researchers to ensure that, by current scientific standards and known methods, no one will be able to identify you from the information we share. Despite these measures, we cannot guarantee anonymity of your personal data.

Can I be removed from the research without my OK?
The person in charge of the research study or the sponsor can remove you from the research study without your approval. Possible reasons for removal include safety issues.

We will tell you about any new information that may affect your health, welfare, or choice to stay in the research.

What else do I need to know?
If you become ill or are injured because of these study procedures, you should seek medical treatment through your doctor or treatment center of choice. You should promptly tell the study doctor about any illness or injury.

The hospital. University or the researchers will not pay for medical care required because of a bad outcome resulting from your participation in this research study. This does not keep you from seeking to be paid back for care required because of a bad outcome.

Permission to Take Part in a Human Research Study
Do not sign this consent if today's date is later than the stated expiration date above.

If you agree to take part in this research study, we will pay you $25.00 for each visit for your time and effort. You will receive $600.00 if you complete the study and attend all visits. If you do not complete the study, we will pro-rate your payment based on $25.00 per visit.

If ClinCard is not available at the time you are enrolled in the study, you will be paid by check. The Accounting Services at Northwestern University will be given your name, address, and Social Security Number in order to issue a check for your study participation. Study payments are considered taxable income and reportable to the IRS. A Form 1099 will be sent to you if your total payments are $600 or more in a calendar year.

You will be paid $25 for each session that you attend (total of $600.00). These funds are provided to help support you with time and travel associated with your participation. The Shirley Ryan AbilityLab will issue you a ClinCard, which is a specially designed debit card for clinical research. Once a visit that qualifies for compensation is completed, funds will be approved and loaded onto your card. The funds will be available within 1 day after being loaded and can be used at your discretion. You will be issued one card for the duration of your participation. If your card is lost or stolen, please call (866) 952-3795 or ask a coordinator for a replacement ClinCard.

Fees are incurred if used at an ATM (fees vary by location). However, if the card is used for in-store or online purchases via credit or debit, there are no associated fees and no expiration date.

Please be advised: Inactivity on the card for more than 3 months will incur a monthly fee. However, as long as there is activity on the card within 3 months (funds are added or a transaction is completed), the month period will reset and no monthly fee will be assessed.

If you do incur a monthly fee, please contact Greenphire Support at the number on the back of your card and they will reverse the fee. See "Tips for Using the Attached ClinCard" for more information.

The Finance Department at the Shirley Ryan AbilityLab will be provided with your information, including your Social Security Number, in order to issue payment for your study participation. Study payments are considered taxable income and reportable to the Internal Revenue Service (IRS). An IRS Form 1099 will be sent to you if your total payments are $600 or more in a calendar year.

Payment for Screening: For the day of screening only, you will not be issued a ClinCard. You will be issued the Stored Value Card (VISA), which is a type of bank debit card with a specific dollar value programmed into it. Once a visit that qualifies for compensation is completed, funds will be approved and loaded onto your card.

You will need to set a PIN to use the card at an ATM. Using the PNC automated service number and Account Access Code provided on the card, follow the prompts to establish a PIN. You may also call this number to obtain the current balance on the card and to verify your activity. A fee will be charged to speak to a live operator. This information can also be checked online at

pncprepaidcard.com. Please note that neither PNC nor Northwestern can obtain the PIN if forgotten.

If the card is used at a PNC ATM, there is no fee; however, there will be a $2.50 charge for non-PNC ATM withdrawals. One card will be issued for the duration of your participation. If your card is lost or stolen, please call the study team on the contact information provided on this consent document.

Please be advised: You will incur a fee if the card is not used in 6 months and a monthly fee for each additional month of non-use. However, as long as there is activity (funds are added or card is used), on the card within 6 months the month period will reset and no monthly fee will be assessed. If the card is used at a restaurant, there will be a 20% "hold" above the tab amount. The card will be declined if used at a gas pump. Rather, the card must be physically presented to the gas station attendant.

You may be given access to new inventions that are being developed by the investigator, the study sponsor, or other people involved in the study. Certain laws can make it harder to obtain legal protection for a new invention shared with a study participant, unless the study participant agrees to keep information about the invention confidential. You agree to keep confidential information you may receive about new inventions, such as new drugs, new devices, or new methods.

At post-treatment and follow-up, you can examine the results of your own progress. If you want to know the results of the study in its entirety, you must ask Dr. Mary Ellen Stoykov (312) 503-3106 or mstoykov@sralab.org.

HIPAA Authorization
We are committed to respect your privacy and to keep your personal information confidential. When choosing to take part in this study, you are giving us the permission to use your personal health information that includes health information in your medical records and information that can identify you. For example, personal health information may include your name, address, phone number or social security number. Your health information we may collect and use for this research includes:

- All information in a medical record
- Results of the arm tests
- Results of the TMS and the grip termination task
- Medical history

This consent expires on September 29, 2019. After this date, Northwestern University may not gather new information about you, use or disclose your personal health information collected in this study for any purpose other than the research study described in this consent unless Northwestern University obtains permission to do so from you.

The following clinical providers may give the researchers information about you: all current and previous health care providers, including but not limited to the Shirley Ryan AbilityLab

(SRALAB), Northwestern Medical Group (NMG), Northwestern Memorial Hospital (NMH), and Northwestern Lake Forest Hospital (NLFH).

Once we have the health information listed above, we may share some of this information with the following offices or entities outside of Northwestern University and its clinical partners (or affiliates): the Northwestern University Institutional Review Board Office and Office for Research Integrity; the US Office of Research Integrity; the US Office for Human Research Protections; the US Food and Drug Administration.

Any research information shared with outside entities will not contain your name, address, telephone or social security number or any other personal identifier unless disclosure of the identifier is necessary for review by such parties or is required by law or University policy [except that such information may be viewed by the Study sponsor and its partners or contractors at the Principal Investigator's office].

The following entities may receive your health information:

- Authorized members of the Northwestern University and the Shirley Ryan AbilityLab workforce, who may need to see your information, such as administrative staff members from the Office for Research, Office for Research Integrity and members of the Institutional Review Board.
- Clinical affiliates, including but not limited to the Shirley Ryan AbilityLab (SRALAB), Northwestern Medical Group (NMG), Northwestern Memorial Hospital (NMH), Northwestern Lake Forest Hospital (NLFH), and the Ann & Robert H. Lurie Children's Hospital of Chicago (Lurie Children's). Your participation in this clinical trial may be tracked in an electronic database and may be seen by investigators running other trials that you are enrolled in and by your healthcare providers.
- Other University research centers and University contractors who are also working on the study,
- Study monitors and auditors who make sure that the study is being done properly,
- Government agencies and public health authorities, such as the Food and Drug Administration (FDA) and the Department of Health and Human Services (DHHS).

Those persons who get your health information may not be required by Federal privacy laws (such as the Privacy Rule) to protect it. Some of those persons may be able to share your information with others without your separate permission.

The results of this study may also be used for teaching, publications, or for presentation at scientific meetings.

Unless you revoke your consent, it will expire at the end of the research study

Although you may revoke consent to participation in this research at any time and in any format, you must revoke authorization for use or disclosure of your health information in writing. To revoke your authorization, write to:

PI's Name: Daniel Corcos, PhD
Institution: Northwestern University

Permission to Take Part in a Human Research Study
Do not sign this consent if today's date is later than the stated expiration date above.

Department: Physical Therapy and Human Movement Science
Address: 645 North Michigan Avenue, Chicago IL 60611

You do not have to authorize the use or disclosure of your health information; however, you will not be allowed to take part in this research study. If you do not authorize the use or disclosure of your health information, it will not affect your treatment by health care providers, or the payment or enrollment in any health plans, or affect your eligibility for benefits.

A copy of this signed consent document, information about this study, and the results of any test or procedure done may be included in your medical records and may be seen by your insurance company.

Optional Elements:
The following research activities are optional, meaning that you do not have to agree to them in order to participate in the research study. Please indicate your willingness to participate in these optional activities by placing your initials next to each activity.

I agree	I disagree	
_______	_______	The researcher may audio or video record me to aid with data analysis. The researcher will not share these recordings with anyone outside of the immediate study team.
_______	_______	The researcher may audio or video record me for use in scholarly presentations or publications. My identity may be shared as part of this activity, although the researcher will attempt to limit such identification. I understand the risks associated with such identification.
________	________	The researcher may audio or video record me for use on publically marketed television programs for the purpose of recruitment. My identity may be shared as part of this activity, although the researcher will attempt to limit such identification. I understand the risks associated with such identification.
________	_______	The researcher may contact me in the future to see whether I am interested in participating in other research studies by the Principal Investigator of this study.

Your signature documents your permission to take part in this research. You will be provided a copy of this signed document.

____ ____________________
Signature of Participant Date

____
Printed Name of Participant

Permission to Take Part in a Human Research Study

Do not sign this consent if today's date is later than the stated expiration date above.

____ __________________
Signature of Person Obtaining Consent Date

____
Printed Name of Person Obtaining Consent
