# Supplementary material for: Bilateral upper extremity motor priming (BUMP) plus task-specific training for severe, chronic upper limb hemiparesis: study protocol for a randomized clinical trial
Source: Trials. 2022 Jun 22;23:523. doi: 10.1186/s13063-022-06465-9 (PMC9214193; doi:10.1186/s13063-022-06465-9)
Supplement: Supplementary file 3 — Additional file 3. Original ethics approval document. Approval of new study from the institutional review board office of Northwestern University. [file 13063_2022_6465_MOESM3_ESM.pdf]

Institutional Review Board Office
Northwestern University

Biomedical IRB
750 North Lake Shore Drive
Rubloff Building, Suite 700
Chicago, Illinois 60611
312-503-9338

Social and Behavioral Sciences IRB
600 Foster Street
Chambers Hall, Second Floor
Evanston, Illinois 60208
847-467-1723

APPROVAL OF NEW STUDY

DATE: November 3, 2017

TO: Dr. Daniel Corcos

FROM: Office of the IRB

APPROVAL DATE: 10/26/2017

EFFECTIVE DATE: 11/3/2017

EXPIRATION DATE: 9/27/2018

The Northwestern University IRB reviewed and approved the submission described below:

Type of Submission:	Initial Study
Review Level:	Committee
Expedited Category:	N/A
Title of Study:	Bilateral Priming plus Task Specific Training for Severe Upper Limb Hemiparesis
Principal Investigator:	Daniel Corcos
IRB ID:	STU00205857
Funding Source:	Name: NIH - this is being submitted in response to a request for Just In Time information, Funding Source ID: 1 R01 HD091492-01
IND, IDE, or HDE:	None
Documents Reviewed:	• Fugl Myer Upper Extremity, Category: Data Collection Tools;<br>• COPM.pdf, Category: Data Collection Tools;<br>• TMS screening, Category: Data Collection Tools;<br>• 3942767_Egrant.pdf, Category: Sponsor Attachment;<br>• Consent, Category: Consent Form;<br>• ARAT, Category: Data Collection Tools;<br>• CAHAI manual, Category: Data Collection Tools;<br>• Madhavan CITI, Category: Training Documents;<br>• UIC IAA, Category: Executed Agreements;<br>• Protocol_Bilateral Priming Plus Task Specific Training for Severe Upper Limb Hemiparesis, Category: IRB Protocol;<br>• CAHAI Score Sheet, Category: Data Collection Tools;<br>• Correspondence_for_STU00205857.doc, Category: Correspondence.
Special Determination(s):	The panel determined the study device to be a non-significant risk device. The study meets the criteria for the abbreviated IDE requirements under 21 CFR 812.2(b).

In conducting this study, you are required to follow the requirements listed in the Northwestern University (NU) Investigator Manual (HRP-103), which can be found by navigating to the IRB Library within the eIRB+ system.

NU IRB approval does not constitute or guarantee institutional approval and/or support. Investigators and study team members must comply with all applicable federal, state, and local laws, as well as NU Policies and Procedures, which may include obtaining approval for your research activities from other individuals or entities.

For IRB-related questions, please consult the NU IRB website at http://irb.northwestern.edu. For general research questions, please consult the NU Office for Research website at www.research.northwestern.edu.
