# Supplementary material for: Bilateral upper extremity motor priming (BUMP) plus task-specific training for severe, chronic upper limb hemiparesis: study protocol for a randomized clinical trial
Source: Trials. 2022 Jun 22;23:523. doi: 10.1186/s13063-022-06465-9 (PMC9214193; doi:10.1186/s13063-022-06465-9)
Supplement: Supplementary file 4 — Additional file 4. Model release. Model release form from Exsurgo Rehabilitation, Ltd., singed by the photographer R. Little. [file 13063_2022_6465_MOESM4_ESM.pdf]

Exsurgo Rehab Ltd - Model Release Form

PHOTOGRAPHER: R LITTLE

MODEL: Michael John Brown.

MODEL'S MAILING ADDRESS: 61 Robinson Road Whitianga 3510

MODEL'S EMAIL ADDRESS: whitibrown@vodafone.co.nz

MODEL'S PHONE NUMBER: 021 995323

PHOTOGRAPHS/VIDEOS TAKEN ON (date): 13th Nov 15.

AT (location): AUCKLAND CITY HOSPITAL

I hereby assign full copyright of these photographs/videos to Exsurgo Rehab Ltd (and related representatives and assignees) together with the right of reproduction either wholly or in part.

I grant to Exsurgo Rehab Ltd or licensees or assignees the permission to the above-mentioned photographs/video either separately or together, either wholly or in part, the perpetual and irrevocable and unrestricted right to use and publish photographs/videos of me, or where I may be included for editorial trade, product advertising and such other business purpose in any manner and medium.

Exsurgo Rehab Ltd and licensees or assignees may have unrestricted use of these images for whatever purpose, including advertising, with any retouching or alteration without restriction.

I agree that Exsurgo Rehab Ltd or any person authorized by or acting on behalf may use the above mentioned photographs/videos or any reproductions of them for any advertising purposes or for the purpose of illustrating any wording, however that no such wording shall be considered to be attributed to me personally unless agreed separately.

I undertake not to prosecute or to institute proceedings, claims or demands against either Exsurgo Rehab Ltd, the Photographer their agents in respect of any usage of the above mentioned photographs/videos. I hereby release the Exsurgo Rehab Ltd and the photographer named above from all claims and liability relating to images, video or photographs taken of me.

I have read this model release form carefully and fully understand its meanings and implications.

I agree to my name being used in association with the above mentioned images Y / N (Y circled)

Signed: MJ Brown **date:** 13/11/2015.

Important: If the Model is under 18 year of age, a parent or legal guardian must also sign
